# Correction to: Serological and Molecular Detection of Toxoplasma Gondii among Slaughtered Domestic Ruminants in Gondar Town, Northwest Ethiopia

**DOI:** 10.1002/vms3.70733

**Published:** 2025-12-20

Yirsa, T., Z. Seyoum, M. Dagnaw, N. Berhane. 2025. “Serological and Molecular Detection of Toxoplasma Gondii Among Slaughtered Domestic Ruminants in Gondar Town, Northwest Ethiopia”. *Veterinary Medicine and Science*. 11 no. 6:e70647, https://doi.org/10.1002/vms3.70647.

**Description of error**:

In the published article, the authors’ affiliations were incorrectly listed as follows:

Tsedalu Yirsa^1^*, Zewdu Seyoum^2^, Mequanente Dagnaw^3^, Nega Berhane^3^

^1^ Department of Veterinary Medicine, College of Agriculture, Woldia University, Woldia, Ethiopia

^2^ Department of Veterinary Paraclinical Studies, College of Veterinary Medicine and Animal Sciences, University of Gondar, Gondar, Ethiopia

^3^ Department of Epidemiology and Biostatistics, Department of Medical Biotechnology, Institute of Public Health, Institute of Biotechnology, University of Gondar, Gondar, Ethiopia

**The correct affiliations are**:

Tsedalu Yirsa^1^*, Zewdu Seyoum^2^, Mequanente Dagnaw^3^,⁴, Nega Berhane⁴

^1^ Department of Veterinary Medicine, College of Agriculture, Woldia University, Woldia, Ethiopia

^2^ Department of Veterinary Paraclinical Studies, College of Veterinary Medicine and Animal Sciences, University of Gondar, Gondar, Ethiopia

^3^ Department of Epidemiology and Biostatistics, Institute of Public Health, College of Medicine and Health Sciences, University of Gondar, Gondar, Ethiopia

⁴ Department of Medical Biotechnology, Institute of Biotechnology, University of Gondar, Gondar, Ethiopia

**Correspondence**: Tsedalu Yirsa, Email: tsedyirsa@gmail.com

We apologize for this error.

